# Transcriptome-scale similarities between mouse and human skeletal muscles with normal and myopathic phenotypes

**DOI:** 10.1186/1471-2474-7-23

**Published:** 2006-03-07

**Authors:** Alvin T Kho, Peter B Kang, Isaac S Kohane, Louis M Kunkel

**Affiliations:** 1 Children's Hospital Informatics Program, Harvard-MIT Division of Health Sciences and Technology, Cambridge, Massachusetts, USA; 2 Program in Genomics, Children's Hospital Boston and Harvard Medical School, Boston, Massachusetts, USA; 3 Department of Neurology, Children's Hospital Boston and Harvard Medical School, Boston, Massachusetts, USA; 4 Howard Hughes Medical Institute, Children's Hospital Boston, Boston, Massachusetts, USA

## Abstract

**Background:**

Mouse and human skeletal muscle transcriptome profiles vary by muscle type, raising the question of which mouse muscle groups have the greatest molecular similarities to human skeletal muscle.

**Methods:**

Orthologous (whole, sub-) transcriptome profiles were compared among four mouse-human transcriptome datasets: (M) six muscle groups obtained from three mouse strains (wildtype, *mdx*, *mdx*^5*cv*^); (H1) biopsied human quadriceps from controls and Duchenne muscular dystrophy patients; (H2) four different control human muscle types obtained at autopsy; and (H3) 12 different control human tissues (ten non-muscle).

**Results:**

Of the six mouse muscles examined, mouse soleus bore the greatest molecular similarities to human skeletal muscles, independent of the latters' anatomic location/muscle type, disease state, age and sampling method (autopsy versus biopsy). Significant similarity to any one mouse muscle group was not observed for non-muscle human tissues (dataset H3), indicating this finding to be muscle specific.

**Conclusion:**

This observation may be partly explained by the higher type I fiber content of soleus relative to the other mouse muscles sampled.

## Background

Animal models of human diseases are used extensively to study basic disease processes and test potential therapies. Even though these proxies have ethical and practical advantages, they generally do not completely recapitulate the human disease phenotype. For example, *mdx *and *mdx*^5*cv *^mice have mutations in the dystrophin gene mirroring the genetic defect of human Duchenne muscular dystrophy (DMD) [1-4], yet they experience milder muscle degeneration than DMD patients [3,5,6]. Consequently, extrapolating findings from a mouse model to human disease can have limitations.

Different skeletal muscle groups are dissimilarly affected in muscular dystrophies, suggesting inherent molecular and physiological differences among muscle groups. This raises the question of whether one type of mouse muscle more accurately represents particular (myopathic) characteristics in a given human muscle type than another. Based on gross histology, skeletal muscles differ in at least four parameters: bulk, length, fiber architecture and fiber type proportions. It is not immediately clear how to prioritize these for cross species comparisons. Recent transcriptome studies of different skeletal muscle groups in humans [7-10] and mice [11-16] raise the related question of whether and how muscle groups are related from a molecular perspective, within and across species.

This study investigates the similarities between mouse and human skeletal muscle gene expression profiles at the level of the whole transcriptome and four distinct sub-transcriptomes. Of the six mouse muscle groups considered, soleus was found to be most similar to all human muscles tested, independent of the latters' anatomic location/muscle type, disease state, age, and sampling method (autopsy versus biopsy). These observations were consistent regardless of the sub-transcriptome used to characterize each sample profile.

## Methods

### Transcriptome datasets (M, H1, H2, H3)

The gene expression datasets originate from four separate, previously-published skeletal muscle studies (one on mouse and three on humans) performed on the Affymetrix GeneChip platform (Table).

Dataset M (mouse, n = 36) was derived from six different skeletal muscle groups: diaphragm, extensor digitorum longus, gastrocnemius, quadricep, soleus, and tibialis anterior of eight-week-old male mice. The mice represented three genetic strains: wildtype (C57BL/10SnJ), *mdx *(C57BL/10ScSn-Dmd^mdx/J^), and *mdx*^5*cv *^(B6Ros.Cg-Dmd^mdx-5cv^), with their transcriptome profiles assayed in duplicate on the Affymetrix U74Av2 platform [17,18].

Dataset H1 (human, n = 24) consists of transcriptome profiles of surgically-biopsied quadriceps from 12 control and 12 DMD subjects assayed on the Affymetrix U95Av2 platform [8,19].

Dataset H2 (human, n = 32) contains transcriptome profiles of four different human muscle groups: deltoid, gastrocnemius, quadriceps, and tibialis anterior obtained at autopsy from each of 8 individuals – three pediatric and five geriatric – assayed on the Affymetrix U133A platform [20].

Dataset H3 (human, n = 24) contains transcriptome profiles of 12 distinct normal organ-tissues pooled from 10–25 individuals – bone marrow, brain, heart, liver, lung, kidney, skeletal muscle, pancreas, prostate, spinal cord, spleen, and thymus – assayed on the Affymetrix U95Av2 microarrays in duplicate [21,22].

### Mouse-human gene orthologues

Curated and predicted National Center for Biotechnology Information (NCBI) Entrez-identified mouse-human orthologue gene pairs were obtained from NCBI HomoloGene, (data freeze October 07, 2005) [23]. To ensure a one-to-one and well-defined mapping, cross-species matches were restricted to those with the highest reciprocal percentage sequence homology. For instance, the human gene *ACAT2 *(EntrezID 39) has two distinct mouse orthologues: *Acat2 *(87.12% sequence homology, EntrezID 110460) and *Acat3 *(85.10% sequence homology, EntrezID 224530). *Acat2 *was considered the human *ACAT2*'s unique mouse-orthologue since it had the higher percentage homology with *ACAT2*.

The Affymetrix GeneChip platforms contain the following number of Entrez-identified genes (data freeze May 17, 2005): U74Av2, 8,894 mouse genes; U95Av2, 8,978 human genes; U133A, 12,963 human genes. Between the mouse U74Av2 and human U95Av2 platforms, there exist 5,306 mouse-human orthologue pairs. Similarly, mouse U74Av2 and human U133A platforms have 6,547 orthologue pairs.

### Mathematical analyses

The primary gene set for cross-species analyses are the 5,306 orthologue gene pairs between the mouse Affymetrix U74Av2 (dataset M) and human Affymetrix U95Av2 (dataset H1 and H3) platforms. Of these 5,306 genes, 5,288 (99.7%) are shared in common with the 6,547 U74Av2/U133A orthologue pairs, and were thus used in cross-species analyses between datasets M and H2 (Affymetrix U133A). Each sample gene expression profile is represented as algebraic *N*-vector signifying the reported expression intensities for *N *(>0) distinct genes measured in that sample.

Principal component analysis (PCA) was used to obtain the subsets of genes that were dominant contributors to the global sample variation in datasets M and H1, respectively [see Additional file 1] [see Additional file 2] [see Additional file 3] [24-27]. We define the ***g*_*j*_**'s with |*a*_*j*_| > 0.03 in any one of principal components 1–3 (PC1-3) following PCA of datasets M (271 genes) and H1 (234 genes) to be the dominant contributors to global sample variation for datasets M and H1 respectively. Eighty-six genes are common to both sets.

Linear (Pearson) correlation was used to quantify the linear similarity between mouse and human samples [24]. Each mouse or human sample is a gene expression profile vector of length *N*, with the *j*^th ^mouse vector component being orthologous to the *j*^th ^human vector component. When calculating the mouse-human profile correlations at the whole transcriptome-scale, *N *= 5,306, the total number of orthologue gene pairs between the mouse U74Av2-human U95Av2 platforms; and *N *= 234 (respectively, *N *= 271 – see preceding paragraph) when calculating the mouse-human profile correlations based on the subset of genes that are dominant contributors to global sample variance in dataset H1 (respectively, dataset M).

The non-parametric Wilcoxon (Mann-Whitney) rank-sum test [28] was used to assess the probability that the distribution of linear correlations of human skeletal muscle to mouse soleus is similar to the distribution of linear correlations of human skeletal muscle to mouse non-soleus muscles. The underlying expression data distributions were non-Gaussian, thus a parametric test was not used.

## Results

### Whole transcriptome scale correlations between mouse and human skeletal muscles

Whole transcriptome scale (5,306-gene profile) similarities between skeletal muscle datasets M (mouse) and H1 (human) were investigated. Linear correlation was used as a measure of similarity. Two additional human transcriptome datasets were considered as *in silico *positive controls: (H2) transcriptome profiles from four skeletal muscle groups of eight human subjects from autopsies measured on the Affymetrix U133A platform [20]; and (H3) transcriptome profiles from 12 distinct human tissues (two muscle, ten non-muscle) [21] measured on the same microarray platform as H1.

**Figure 1:**
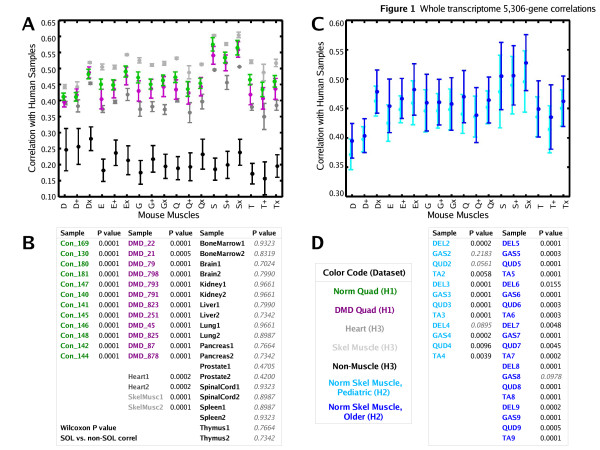
Correlations of human transcriptome profiles relative to homologous mouse muscle profiles. All samples are characterized by their 5,306-gene whole transcriptome profile. (A) Human linear correlation group averages ± one standard deviation error bars relative to each of the six mouse muscle groups × three genetic strains from dataset M. The five human groups are color coded: control (green) and DMD (magenta) quadriceps from dataset H1, pooled skeletal muscle (light gray), heart (dark gray), and tissue not primarily composed of muscle (black) from dataset H3. (B) For each human sample, the likelihood (Wilcoxon rank-sum) that there is no difference in the human sample's correlations to soleus versus to non-soleus mouse muscle profiles is shown. (C, D) Similar to (A, B) except that relative to dataset M, the human samples compared are four different skeletal muscle groups from each of eight normal subject autopsies of dataset H2, forming two color-coded groups: pediatric (light blue) and older (dark blue) samples. Among mouse muscles, human muscle sample groups were consistently most correlated with the mouse soleus.

Linear correlations between 5,306-gene transcriptome profiles of each sample from dataset M to each sample from dataset H1 were computed. The human correlation group averages and standard deviations relative to each of the six mouse muscle groups × three genetic strains are shown in Figure 1A–B. All 24 human quadriceps samples had a significantly higher correlation to mouse soleus than to non-soleus mouse muscles (*p *< 0.02, Wilcoxon), independent of mouse genetic background and human myopathic condition.

To determine whether the higher correlation of human quadriceps to mouse soleus could be reproduced with other human muscles, similar correlation analyses were performed with human datasets H2 and H3 relative to dataset M. Cross-species linear correlation analyses of the 5,288-gene pairs selected between datasets M and H2 showed a significantly higher correlation in 28 of the 32 human samples to mouse soleus (*p *< 0.02, Wilcoxon) than to non-soleus mouse muscles (Figure 1C–D).

To determine whether the higher correlation of human skeletal muscles to mouse soleus is specific to muscle tissue, similar correlation analyses were performed with the 5,306-gene transcriptome profiles of human tissue not primarily composed of muscle (bone marrow, brain, liver, lung, kidney, pancreas, prostate, spinal cord, spleen and thymus) in dataset H3 relative to dataset M. The non-muscle human profiles did not return a significantly higher correlation to any mouse muscle, whereas the pooled human heart and skeletal muscle profiles in dataset H3 showed a significantly higher correlation to the mouse soleus than to non-soleus mouse muscles (*p *< 0.02, Wilcoxon) (Figures 1A, 1C). Indeed, non-muscle human tissue-to-mouse muscle correlations were significantly lower than human muscle-to-mouse muscle correlations.

### Sub-transcriptome scale correlations between mouse and human skeletal muscles

We identified candidate subsets of genes (by their ontologic category or contribution to sample variation *in silico*) that might underwrite the transcriptomic resemblance of human muscle to mouse soleus. Correlation analyses of mouse-human sample profiles were performed as above, relative to four subsets of the 5,306-gene whole transcriptome separately: ST1, the 733-gene sub-transcriptome (13.8% of the whole transcriptome) recorded by the Gene Ontology Consortium [29] to be integral to cell plasma membrane – note that dystrophin and other sarcolemma-related structural proteins involved in myopathies belong to this ontologic category; ST2, the 271-gene sub-transcriptome (5.1% of the whole transcriptome) of dominant contributors to global sample variance in dataset M; ST3, the 234-gene sub-transcriptome (4.4% of the whole transcriptome) of dominant contributors to global sample variance in dataset H1; and ST4, the 4,188-gene sub-transcriptome complement to ST1-3 as a negative *in silico *control – these are genes neither integral to the cell plasma membrane nor dominant contributors to global sample variance in the muscle datasets M and H1. Twenty-one genes in ST2, and 16 in ST3, belong to the cell plasma membrane category/ST1.

**Figure 2:**
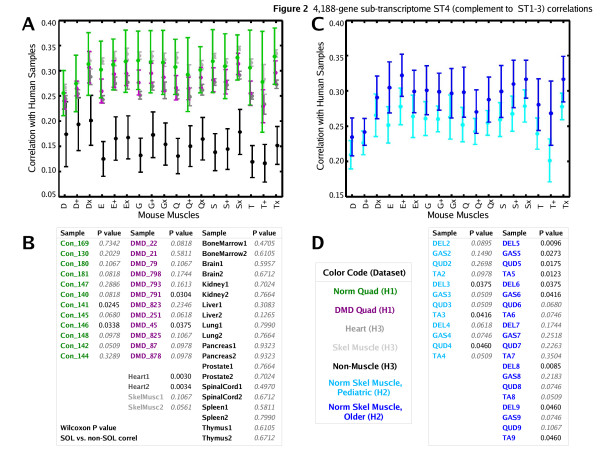
Analogous to Figure 1, correlations of human gene expression profiles relative to homologous mouse muscle profiles, except that samples are characterized as a 4,188-gene profile (ST4) of genes complementary to sub-transcriptomes ST1-3. This is a negative control comparison. Note that human muscle group profiles were not more correlated to any one particular mouse muscle.

Using ST1-3 to characterize sample profiles, we again observed significantly higher correlations of human muscles in datasets H1-3 to mouse soleus than to non-soleus mouse muscles (*p *< 0.02, Wilcoxon) [see Additional file 4] [see Additional file 5] [see Additional file 6]. Similarly, correlations of non-muscle human tissue in dataset H3 to mouse dataset M are lower and do not show a significantly higher correlation to any mouse muscle group – with one exception in the ST3 sample profile characterization where among non-muscle human tissue, brain and spinal cord had significantly higher correlation with mouse soleus than with non-soleus muscle profiles. In contrast, when ST4 was used to characterize sample profiles, all human muscle and non-muscle samples from dataset H1-3 have lower correlations to mouse muscle profiles in dataset M compared to their ST1-3 sample profile characterization, and human muscle profiles show no significant correlation to any mouse muscle group (Figure 2).

### Differential correlations of normal versus DMD human quadriceps to normal mouse skeletal muscle transcriptomes

We investigated whether the myopathic state (specifically DMD) of a human muscle (specifically quadriceps in dataset H1) was reflected in its correlation against wildtype mouse skeletal muscle groups (dataset M).

**Figure 3:**
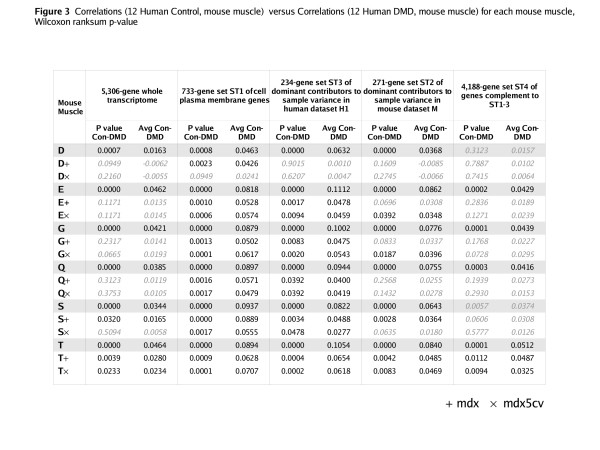
Difference in correlations of control human quadriceps to each mouse skeletal muscle group, and correlations of DMD human quadriceps to each mouse skeletal muscle group – relative to five different sub-transcriptome sample profile characterizations. In other words, Δ(Corr(control human quadriceps, mouse muscle), Corr(DMD human quadriceps, mouse muscle)). A Wilcoxon rank-sum test was used to assess the likelihood of non-difference in these correlations. In all but the ST4 (negative control) sample profile characterization, mouse wildtype muscles were more correlated to control (Con) than to DMD human quadriceps.

Mouse-human correlation analyses were performed between datasets M and H1, relative to the above sample profile characterizations: whole transcriptome and sub-transcriptomes ST1-4. In all except the ST4 characterization, control human quadriceps to wildtype mouse muscle correlations were significantly higher than DMD human quadriceps to wildtype mouse muscle correlations (*p *< 0.02, Wilcoxon) (Figure 3).

## Discussion

All human skeletal muscle samples tested were found to be significantly more correlated to mouse soleus than to the other five non-soleus mouse skeletal muscle groups, independent of the human samples' anatomic location/muscle type, disease state, age and sampling method (autopsy versus biopsy). This observation was both specific to muscle tissue, and consistent even when distinct sub-transcriptomes were used to characterize the mouse-human sample profiles. These *in silico *transcriptome similarities might reflect common/analogous molecular hierarchies modulating these system biologies. From a practical perspective, the approach taken here can be extended to assessing general animal models of human systems *in silico*.

Mouse soleus bears a greater molecular resemblance to several human skeletal muscle types under different conditions (autopsy and biopsy sampling, disease and control) than the other five mouse muscles examined. This suggests that mouse soleus may be one of the better representations of human muscle disease among mouse muscles, to study both disease processes and potential therapies. Besides having a molecular profile that is distinct from the other five mouse skeletal muscle groups [17], soleus also displays histological differences. Soleus has 58% type I fibers, whereas the other muscles have lower type I percentages: extensor digitorum longus and tibalis anterior 0%, gastrocnemius 1–8%, quadriceps 0–45%, diaphragm 10–12% [30-33]. Like mouse soleus, the human autopsy samples examined had a balance of fiber types I and II [20], raising the question of whether the molecular resemblances derive primarily from similarities in fiber type proportions. If this were the case, the genes that most distinguish mouse soleus from other mouse muscles, and most represent its similarities to human muscles, should include a significant number of fiber type specific genes. Consistent with this interpretation, we find that among the ten genes with the highest positive loading in PC2 (the axis of greatest variation between mouse soleus versus other muscle groups) for dataset M, six are differentially expressed between fiber types (rank in parentheses): Myl3 (#2) [34], Tnni1 (#3) [35], Myl2 (#4) [36], Tnnc1 (#5) [37], Mb (#7) [38], and Tnnt (#9) [39]. Two of the others, Atp2a2 (#6) [40] and Idh2 (#10),[41] are involved in energy metabolism and may be expressed at different levels in different fiber types. Curiously, Fhl1 (#8) is differentially expressed between rat soleus and gastrocnemius, but this does not appear to be due to fiber type composition [42]. Myh7 (#1) is primarily expressed in cardiac muscle[43] and has been associated with cardiomyopathy,[44,45] but also appears to be expressed in skeletal muscle[46] Two other parameters that influence muscle function – muscle length and fiber length – do not differ significantly between the five non-diaphragm skeletal muscle groups [30].

Functional studies have demonstrated significant differences in the mechanical properties of different mouse muscles, but the data are somewhat conflicting. One study found that the soleus muscle of dystrophin-utrophin double knockout mice had a greater reduction in twitch force than in extensor digitorum longus and diaphragm [47]. In contrast, isometric contractions followed by stretch contractions in extensor digitorum longus and soleus muscles dissected from control and *mdx *mice resulted in irreversible damage only in *mdx *extensor digitorum longus [48]. The interpretation of this data is unclear, as the findings were identified in different mouse strains. The *mdx *mouse would genetically be expected to replicate the human disease most accurately, but the divergent phenotype compared to human DMD raises doubts about this.

The use of animals to model human disease is complicated by many factors. This is especially true in myopathies where there are many different muscle types, some of which may be more representative of human disease than others. Based on phenotypic severity, *mdx *diaphragm appears to bear the closest relationship to human disease. However, the diaphragm is not an extremity muscle. Among extremity muscles in the *mdx *mouse, soleus might merit special attention in studies of the pathophysiology of the muscular dystrophies.

## Conclusion

1. Mouse soleus bears a closer molecular resemblance than other mouse skeletal muscles to several different human skeletal muscles.

2. This resemblance is consistent for both control and disease human tissue, and is specific for human muscle tissue compared to non-muscle tissue.

3. These results may be due in part to differences in fiber type composition.

## List of abbreviations

DMD, Duchenne muscular dystrophy

H1, H2, H3, human datasets

M, mouse dataset

NCBI, National Center for Biotechnology Information

PC, principal component

PCA, principal component analysis

ST1, ST2, ST3, ST4, sub-transcriptomes

## Competing interests

The author(s) declare that they have no competing interests.

## Authors' contributions

ATK helped conceive the study, performed the data analysis, and drafted a large portion of the manuscript. PBK participated in data interpretation, suggested the biological and therapeutic applications, and helped draft and edit the manuscript. ISK participated in data interpretation. LMK helped conceive the study, participated in data interpretation, and coordinated the overall project. All authors read and approved the final manuscript.

**Table 1:** 

Dataset compositions				
*Dataset*	*Tissue*			

M (mouse)	Muscle: EDL, diaphragm, gastrocnemius, quadriceps, soleus, TA			
H1 (human)	Muscle: quadriceps			
H2 (human)	Muscle: deltoid, gastrocnemius, quadriceps, tibialis anterior			
H3 (human)	Bone marrow, brain, heart, liver, lung, kidney, skeletal muscle, pancreas, prostate, spinal cord, spleen, thymus			

Dataset compositions				

*Dataset*	*Conditions*	*Source*	*# of Samples*	*Affymetrix GeneChip*

M (mouse)	Control, *mdx*, *mdx*^5*cv*^	Immediate autopsy	36	U74Av2
H1 (human)	Control, DMD	Surgical biopsy	24	U95Av2
H2 (human)	Control	Autopsy	32	U133A
H3 (human)	Control	Autopsy?	24	U95Av2

## Pre-publication history

The pre-publication history for this paper can be accessed here:



## Supplementary Material

Additional File 1 Adobe pdf file. Principal component analysis. A mathematical review of principal component analysis. Click here for file

Additional File 2 Adobe pdf file. Principal component analysis (PCA) of mouse dataset M. (A, B) PCA of mouse muscle samples characterized as 5,306-gene whole transcriptome profiles. Sample projections into principal components PC1-2 (A) and PC1-3 (B) planes are shown. The dominant variation (along PC1) is between three skeletal muscle clusters: diaphragm, soleus, and the four remaining muscles. (C, D) Analogous to (A, B), PCA of mouse samples characterized as 271-gene profiles of dominant contributors to whole transcriptome sample variation. Dominant variance contributors are defined to be genes which have absolute loading coefficient exceeding 0.03 in PC1-3 in the PCA of the whole (5,306-gene) transcriptome case (A, B). Click here for file

Additional File 3 Adobe pdf file. PCA of human dataset H1. (A, B) PCA of human muscle samples characterized as 5,306-gene whole transcriptome profiles. Sample projections into principal components PC1-2 (A) and PC1-3 (B) planes are shown. The dominant variation (along PC1) is between control and DMD samples. (C, D) Analogous to (A, B), PCA of human samples characterized as 234-gene profiles of dominant contributors to whole transcriptome sample variation. Dominant variance contributors are defined to be genes which have absolute loading coefficient exceeding 0.03 in PC1-3 in the PCA of the whole (5,306-gene) transcriptome case (A, B). Click here for file

Additional File 4 Adobe pdf file. Correlations of human transcriptome profiles relative to homologous mouse muscle profiles. All samples are characterized by their 733-gene profile of cell plasma membrane genes (ST1). (A) Human linear correlation group averages ± one standard deviation error bars relative to each of the six mouse muscle groups × three genetic strains from dataset M. The five human groups are color coded: control (green) and DMD (magenta) quadriceps from dataset H1, pooled skeletal muscle (light gray), heart (dark gray), and tissue not primarily composed of muscle (black) from dataset H3. (B) For each human sample, the likelihood (Wilcoxon ranksum) that there is no difference in the human sample's correlations to soleus versus to non-soleus mouse muscle profiles is shown. (C, D) Similar to (A, B) except that relative to dataset M, the human samples compared are four different skeletal muscle groups from each of eight normal subject autopsies of dataset H2, forming two color-coded groups: pediatric (light blue) and older (dark blue) samples. Among mouse muscles, human muscle sample groups were consistently most correlated with the mouse soleus. Click here for file

Additional File 5 Adobe pdf file. Correlations of human transcriptome profiles relative to homologous mouse muscle profiles. Here, all samples are characterized by their 271-gene profile of dominant contributors to sample variance in the mouse dataset M (i.e., genes with absolute loadings in PC1-3 exceeding 0.03 (ST2). Click here for file

Additional File 6 Adobe pdf file. Correlations of human transcriptome profiles relative to homologous mouse muscle profiles. Here, all samples are characterized by their 234-gene profile of dominant contributors to sample variance in the human dataset H1 (i.e., genes with absolute loadings in PC1-3 exceeding 0.03 (ST2). Click here for file
