# Supplementary material for: Transcriptome-scale similarities between mouse and human skeletal muscles with normal and myopathic phenotypes
Source: BMC Musculoskelet Disord. 2006 Mar 7;7:23. doi: 10.1186/1471-2474-7-23 (PMC1525166; doi:10.1186/1471-2474-7-23)
Supplement: Additional File 1 — Adobe pdf file. Principal component analysis. A mathematical review of principal component analysis. [file 1471-2474-7-23-S1.pdf]

Supplement 1: Principal Component Analysis

A general microarray dataset can be represented as an N genes (probes) $\times$ M samples matrix, where typically $N >> M$. Call this matrix S = [a_{nm}] with real-value entries a_{nm}. S can be viewed from two heuristically distinct perspectives: (1) M genes in N-dimensional sample space, the microscopic perspective, or (2) N samples in M-dimensional gene space, the macroscopic perspective. For this discussion, we will describe PCA of the latter perspective where the data is visualized as M sample points in N-dimensional gene space. Algebraically, M objects require at most M number of independent features for a well-defined characterization. With S, we have an over-determined system (>> M) where each of the M samples is described by N original features (genes). The objective here is to derive a set of K ($\leq M$) new features from the original features that can equivalently characterize the samples, but in a non-redundant manner. To achieve this objective, the basic observation/motivation is that some of the N original features are "correlated" across the M samples (therefore redundant); and the directions of maximal sample variance in dataset's original feature space form an algebraically independent (thus non-redundant) set of new features for characterizing the samples.

The m-th sample in the original feature (gene) space looks like $\boldsymbol{S_m} = a_{1m}\, \boldsymbol{g_1} + a_{2m}\, \boldsymbol{g_2} + \ldots + a_{Nm}\, \boldsymbol{g_N}$ (m = 1, 2, …, M) – a vector of length N with each vector component a_{nm} denoting the measured expression level of the gene $\boldsymbol{g_n}$ in sample $\boldsymbol{S_m}$. Each gene $\boldsymbol{g_n}$ is a standard basis element or a canonical direction in N-dimensional real space, i.e., $\boldsymbol{g_n}$ is a vector of length N that is zero in every component except the n-th component where it is one. Note that the matrix whose column vectors are ordered $\boldsymbol{g_n}$'s is the $N \times N$ identity matrix $I_{N\times N}$.

The sample variance structures within S are captured by its row-wise $N \times N$ covariance matrix Σ. By basic theorems in linear algebra , the eigenvectors of Σ form an algebraically independent, orthonormal set of vectors of length N, and correspond to directions of maximal sample variance of S. Let $\boldsymbol{p_k}$ denote the eigenvectors of Σ for k = 1, 2, …, $K \leq \min(M, N)$. $\boldsymbol{p_k}$'s may be obtained by standard matrix decomposition methods such as singular value decomposition. $\boldsymbol{p_k}$'s are called principal components of S and are traditionally ordered by descending eigenvalue magnitude. The first principal component $\boldsymbol{p_1}$ is the direction of maximum sample variance in S. The second principal component $\boldsymbol{p_2}$ is the direction of maximum sample variance in the gene space orthogonal to space spanned by the previous principal component, $\boldsymbol{p_1}$. The third principal component $\boldsymbol{p_3}$ is the direction of maximum sample variance in the gene space orthogonal to space spanned by the previous principal components, $\boldsymbol{p_1}$ and $\boldsymbol{p_2}$, and so forth by induction. Each principal component $\boldsymbol{p_k}$ is a linear combination of the original features $\boldsymbol{g_n}$, i.e., $\boldsymbol{p_k} = c_{1k}\, \boldsymbol{g_1} + c_{2k}\, \boldsymbol{g_2} + \ldots + c_{Nk}\, \boldsymbol{g_N}$, where the magnitude of c_{nk} signify the contribution of $\boldsymbol{g_n}$ to this k-th principal component. These $\boldsymbol{p_k}$'s are K new features to replace the N original features ($\boldsymbol{g_n}$'s) for the dataset S.

In this work, we considered every original feature (gene) with contribution $|c_{nk}| > 0.03$ in any of principal components k = 1, 2, 3 (PC1-3), to be a dominant contributors to global sample variation in datasets M and H1 respectively.

Let P be the $N \times K$ matrix whose column vectors are principal components, P = [$\boldsymbol{p_1}\ \boldsymbol{p_2}\ \ldots\ \boldsymbol{p_K}$], $S_{\text{orig\_features}}$ = S the data matrix relative to the original features $\boldsymbol{g_n}$'s, and

$S_{new\_features}$ is the data matrix S relative to (non-redundant) new features $\boldsymbol{p_k}$'s. Then $I_{N\times N}*S_{orig\_features} = P*S_{new\_features}$, and $S_{new\_features} = P^T*S_{orig\_features}$, where P^T is the matrix transpose of P. So every sample $\boldsymbol{S_m}$ in the original features, $\boldsymbol{S_m}^{orig\_features} = a_{1m}\, \boldsymbol{g_1} + a_{2m}\, \boldsymbol{g_2} + \ldots + a_{Nm}\, \boldsymbol{g_N}$ is equivalent to $\boldsymbol{S_m}^{new\_features} = b_{1m}\, \boldsymbol{g_1} + b_{2m}\, \boldsymbol{g_2} + \ldots + b_{Nm}\, \boldsymbol{g_K}$, where $b_{km} = \boldsymbol{S_m}^{orig\_features}*\boldsymbol{p_k} = (a_{1m}\ a_{2m}\ \ldots\ a_{Nm})*\boldsymbol{p_k}$. More generally, left multiplication of any sample vector $\boldsymbol{X}$ of length N by P^T is an affine transformation of $\boldsymbol{X}$– assuming that the components of $\boldsymbol{X}$ are equivalent (homologous) to row features of S.
