# Supplementary figures and images for: Transcriptome-scale similarities between mouse and human skeletal muscles with normal and myopathic phenotypes
Source: BMC Musculoskelet Disord. 2006 Mar 7;7:23. doi: 10.1186/1471-2474-7-23 (PMC1525166; doi:10.1186/1471-2474-7-23)

Supp Figure 2 Transcriptome and sub-transcriptome scale PCA of Dataset M

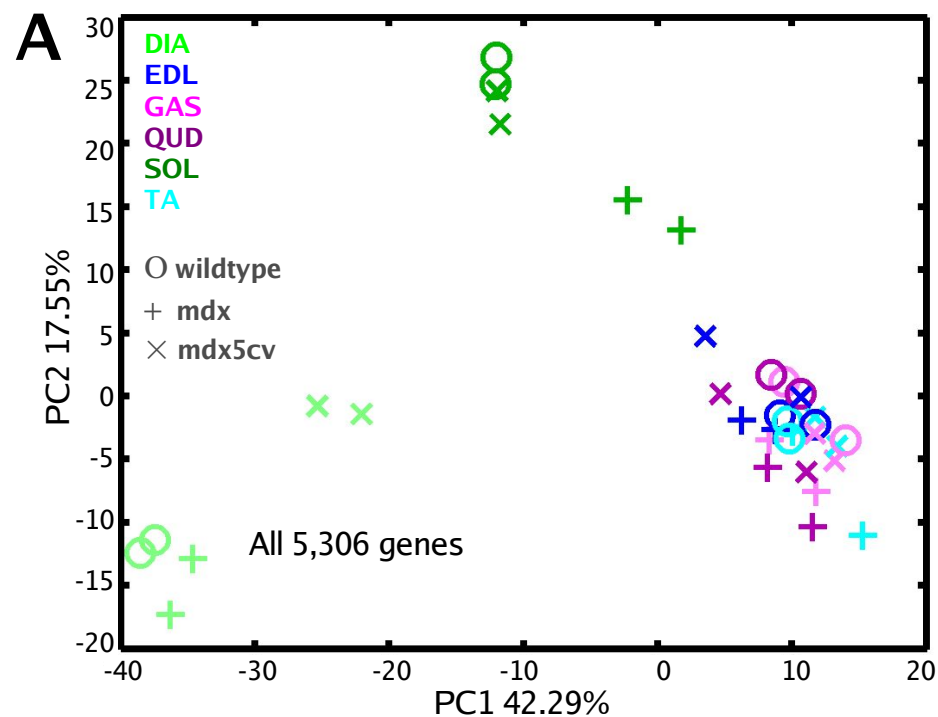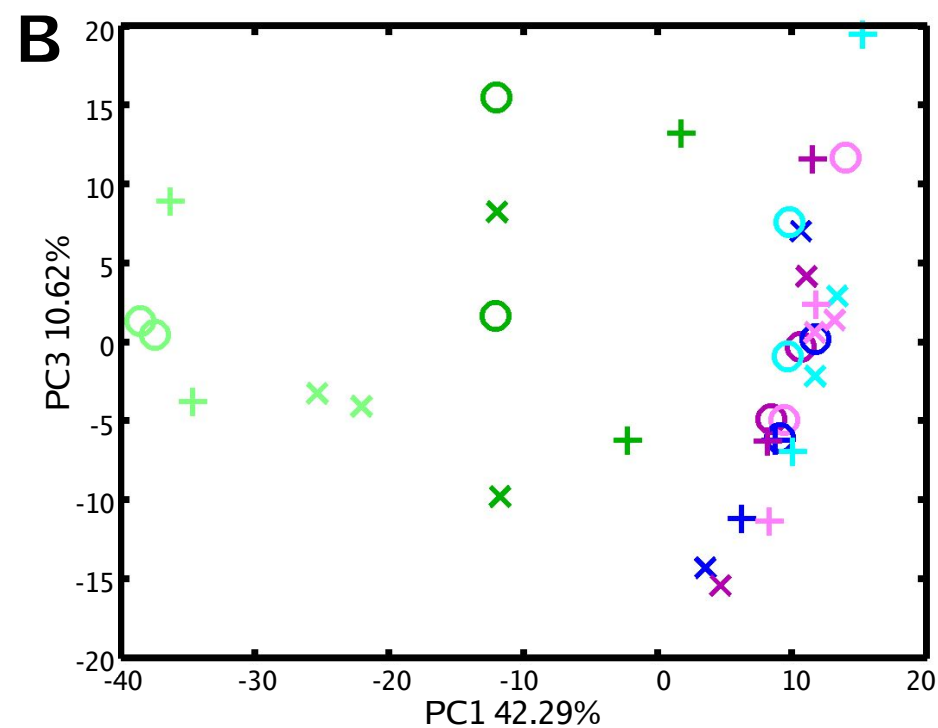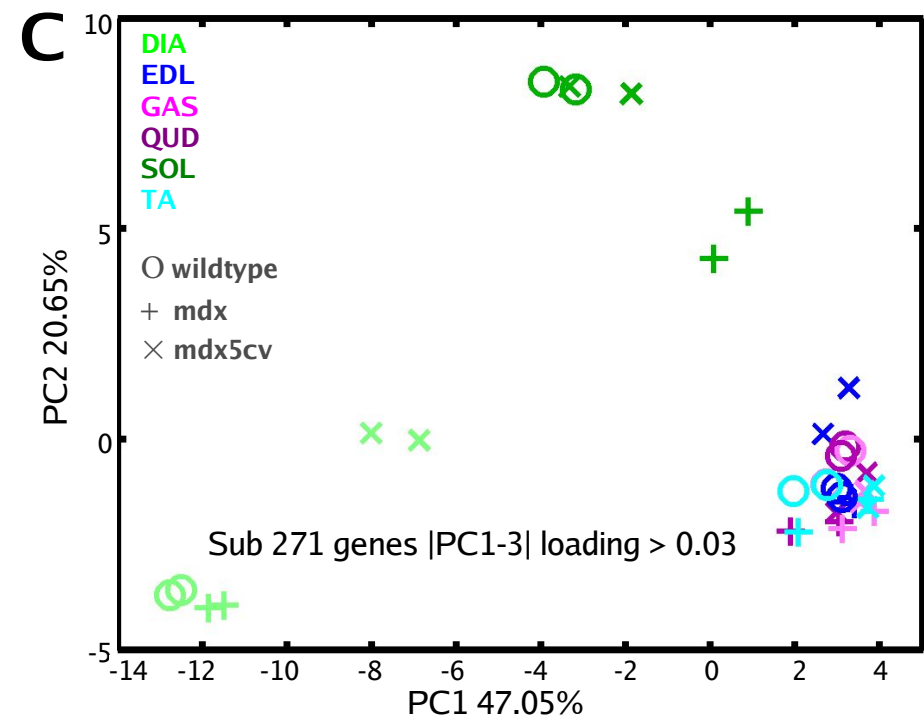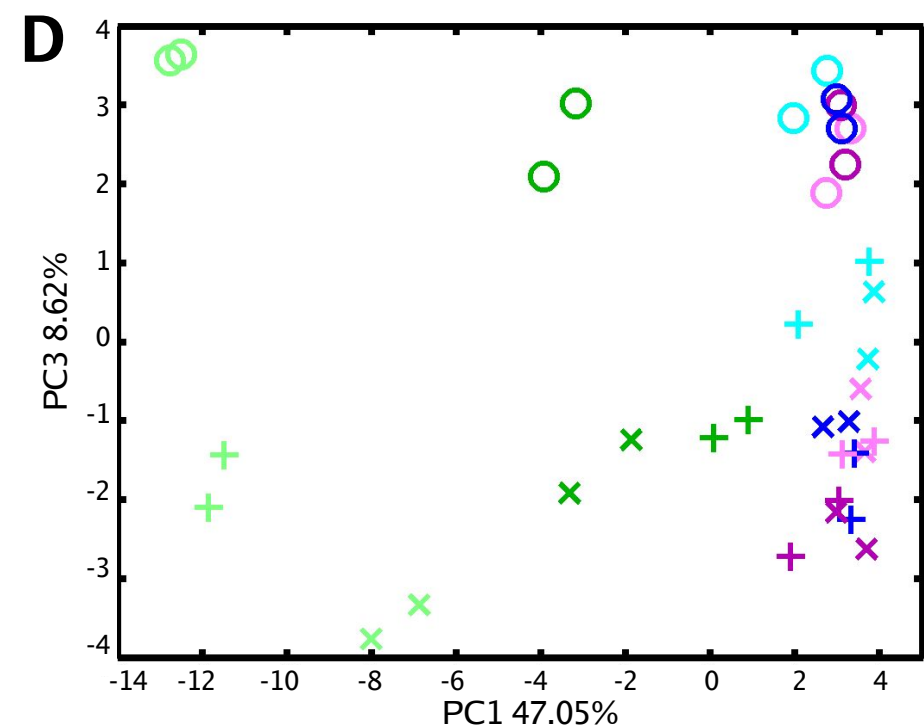

Supplement: Additional File 2 — Adobe pdf file. Principal component analysis (PCA) of mouse dataset M. (A, B) PCA of mouse muscle samples characterized as 5,306-gene whole transcriptome profiles. Sample projections into principal components PC1-2 (A) and PC1-3 (B) planes are shown. The dominant variation (along PC1) is between three skeletal muscle clusters: diaphragm, soleus, and the four remaining muscles. (C, D) Analogous to (A, B), PCA of mouse samples characterized as 271-gene profiles of dominant contributors to whole transcriptome sample variation. Dominant variance contributors are defined to be genes which have absolute loading coefficient exceeding 0.03 in PC1-3 in the PCA of the whole (5,306-gene) transcriptome case (A, B). [file 1471-2474-7-23-S2.pdf]

Supp Figure 3 Transcriptome and sub-transcriptome scale PCA of Dataset H1

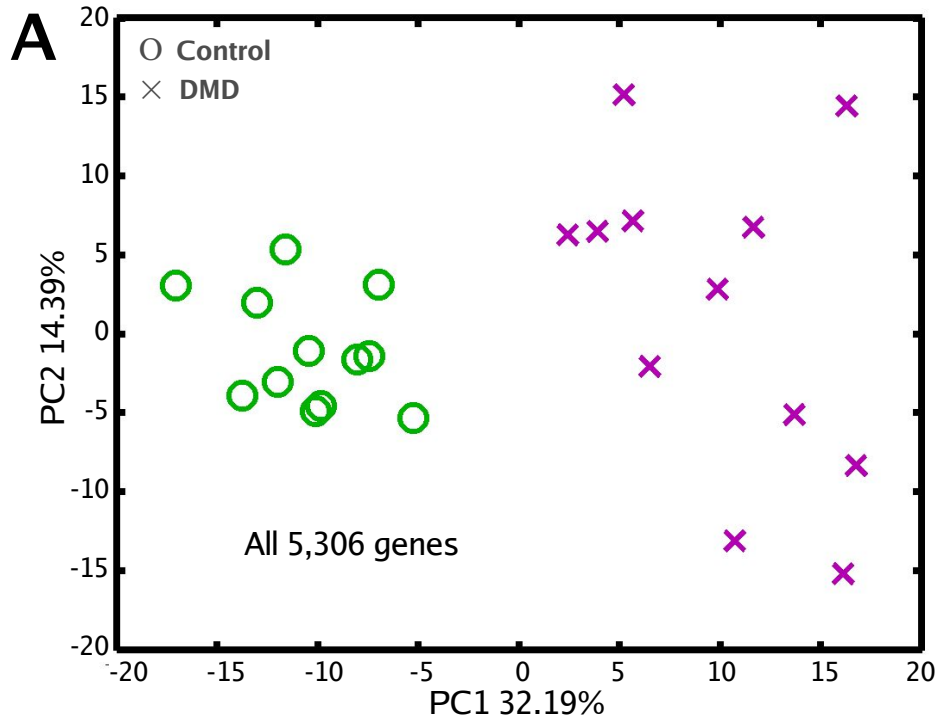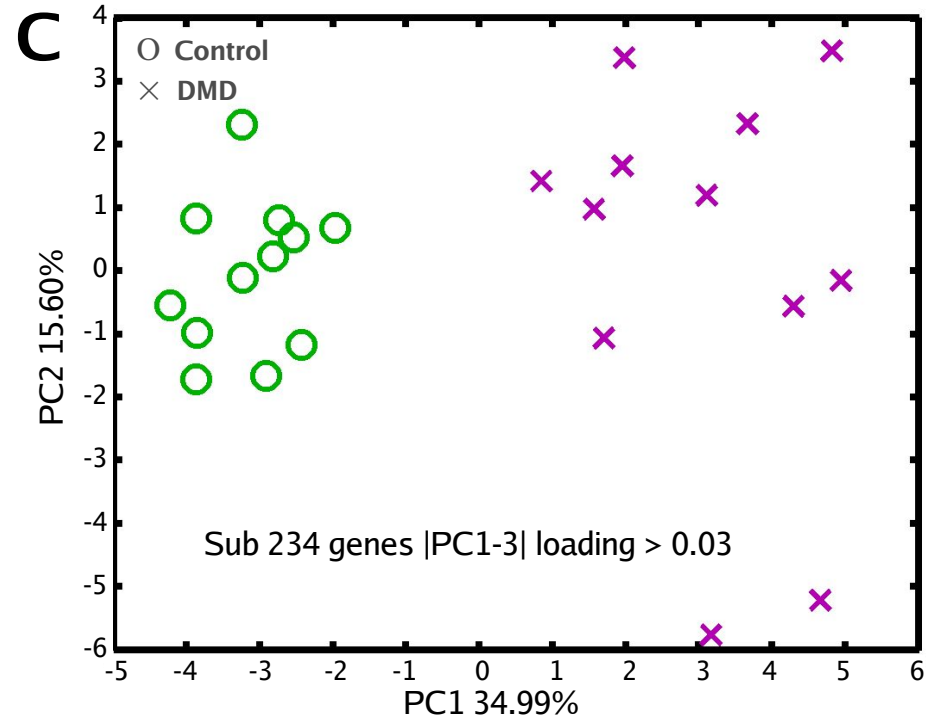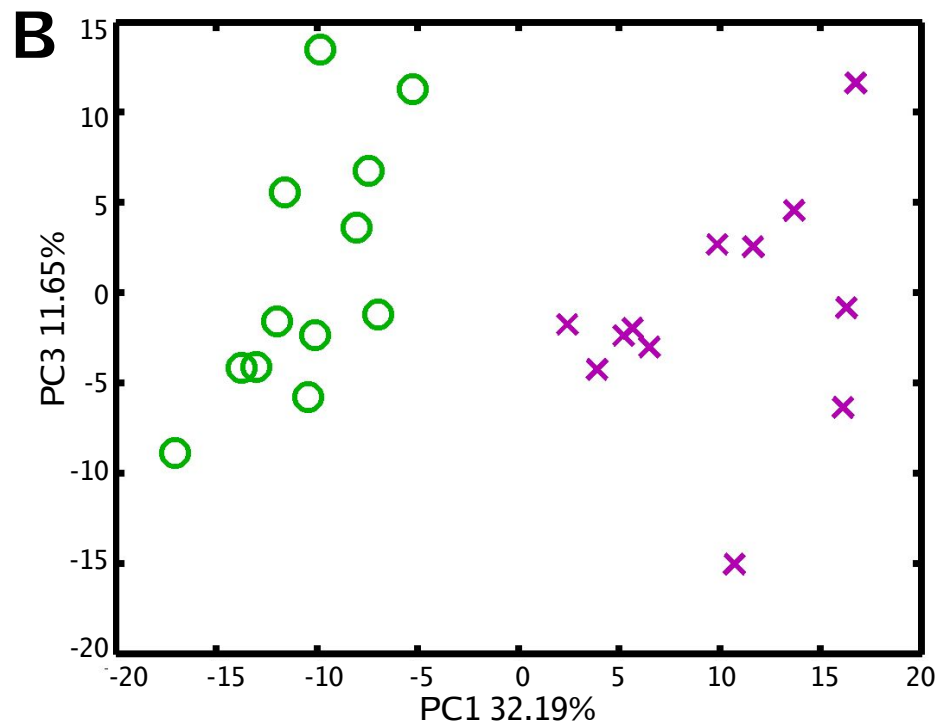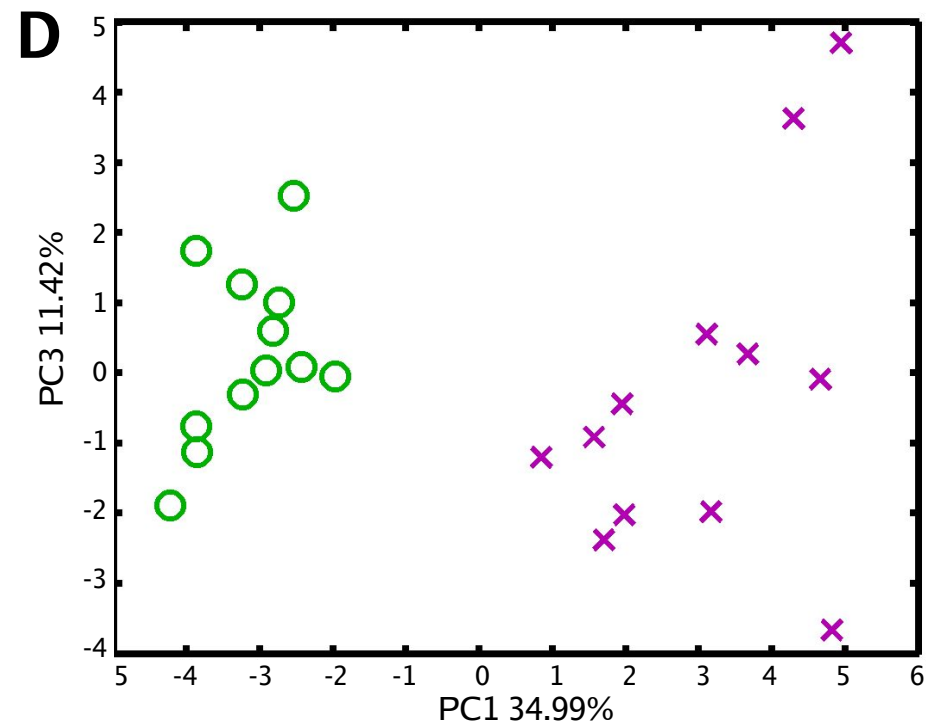

Supplement: Additional File 3 — Adobe pdf file. PCA of human dataset H1. (A, B) PCA of human muscle samples characterized as 5,306-gene whole transcriptome profiles. Sample projections into principal components PC1-2 (A) and PC1-3 (B) planes are shown. The dominant variation (along PC1) is between control and DMD samples. (C, D) Analogous to (A, B), PCA of human samples characterized as 234-gene profiles of dominant contributors to whole transcriptome sample variation. Dominant variance contributors are defined to be genes which have absolute loading coefficient exceeding 0.03 in PC1-3 in the PCA of the whole (5,306-gene) transcriptome case (A, B). [file 1471-2474-7-23-S3.pdf]
