# Supplementary material for: Transcriptome-scale similarities between mouse and human skeletal muscles with normal and myopathic phenotypes
Source: BMC Musculoskelet Disord. 2006 Mar 7;7:23. doi: 10.1186/1471-2474-7-23 (PMC1525166; doi:10.1186/1471-2474-7-23)
Supplement: Additional File 4 — Adobe pdf file. Correlations of human transcriptome profiles relative to homologous mouse muscle profiles. All samples are characterized by their 733-gene profile of cell plasma membrane genes (ST1). (A) Human linear correlation group averages ± one standard deviation error bars relative to each of the six mouse muscle groups × three genetic strains from dataset M. The five human groups are color coded: control (green) and DMD (magenta) quadriceps from dataset H1, pooled skeletal muscle (light gray), heart (dark gray), and tissue not primarily composed of muscle (black) from dataset H3. (B) For each human sample, the likelihood (Wilcoxon ranksum) that there is no difference in the human sample's correlations to soleus versus to non-soleus mouse muscle profiles is shown. (C, D) Similar to (A, B) except that relative to dataset M, the human samples compared are four different skeletal muscle groups from each of eight normal subject autopsies of dataset H2, forming two color-coded groups: pediatric (light blue) and older (dark blue) samples. Among mouse muscles, human muscle sample groups were consistently most correlated with the mouse soleus. [file 1471-2474-7-23-S4.pdf]

**Supp Figure 4** 733-gene plasma membrane sub-transcriptome ST1 correlations

A

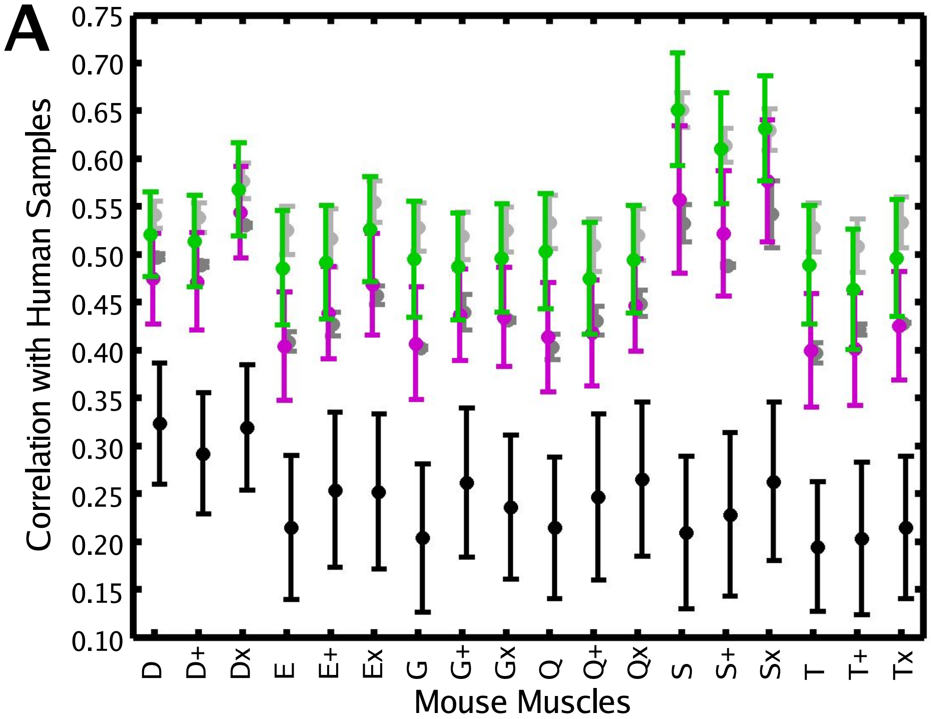


C

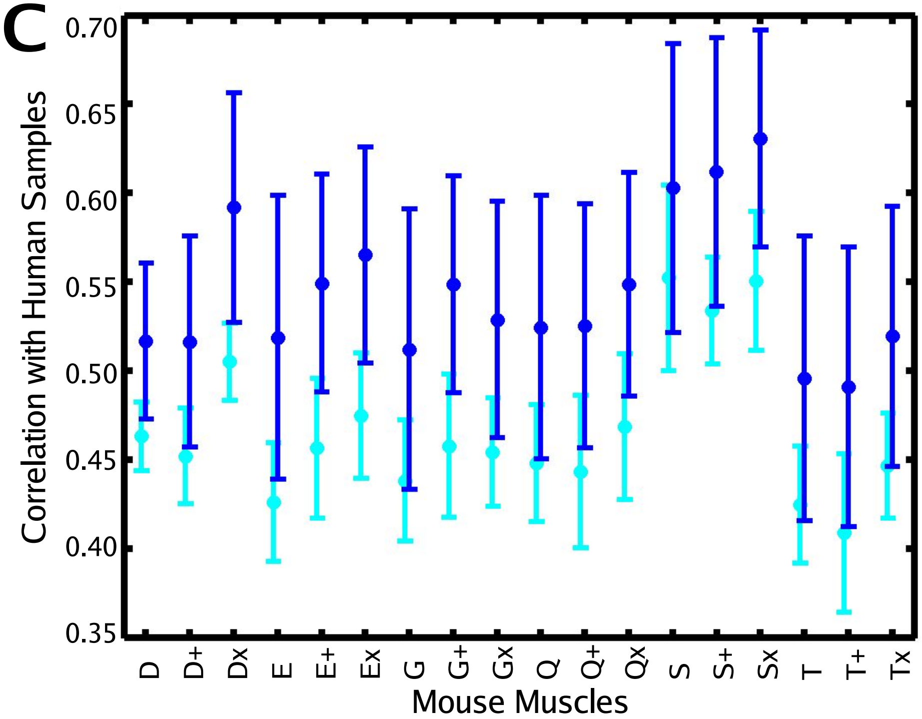


B

| Sample | P value | Sample | P value | Sample | P value |
|---|---|---|---|---|---|
| Con_169 | 0.0001 | DMD_22 | 0.0001 | BoneMarrow1 | *0.3959* |
| Con_130 | 0.0001 | DMD_21 | 0.0058 | BoneMarrow2 | *0.4200* |
| Con_180 | 0.0001 | DMD_79 | 0.0003 | Brain1 | *0.5523* |
| Con_181 | 0.0001 | DMD_798 | 0.0003 | Brain2 | *0.5811* |
| Con_147 | 0.0001 | DMD_793 | 0.0003 | Kidney1 | *0.7024* |
| Con_140 | 0.0001 | DMD_791 | 0.0005 | Kidney2 | *0.6105* |
| Con_141 | 0.0001 | DMD_823 | 0.0003 | Liver1 | *0.7024* |
| Con_145 | 0.0001 | DMD_251 | 0.0004 | Liver2 | *0.6406* |
| Con_146 | 0.0001 | DMD_45 | 0.0002 | Lung1 | *0.8319* |
| Con_148 | 0.0002 | DMD_825 | 0.0005 | Lung2 | *0.8987* |
| Con_142 | 0.0001 | DMD_87 | 0.0003 | Pancreas1 | *0.4970* |
| Con_144 | 0.0001 | DMD_878 | 0.0003 | Pancreas2 | *0.4970* |
| | | | | Prostate1 | *0.7342* |
| | | Heart1 | 0.0015 | Prostate2 | *0.7024* |
| | | Heart2 | 0.0015 | SpinalCord1 | *0.7664* |
| | | SkelMusc1 | 0.0001 | SpinalCord2 | *0.6406* |
| | | SkelMusc2 | 0.0001 | Spleen1 | *0.7664* |
| | | | | Spleen2 | *0.7024* |
| Wilcoxon P value | | | | Thymus1 | *0.3959* |
| SOL vs. non-SOL correl | | | | Thymus2 | *0.3959* |

D

**Color Code (Dataset)**

- Norm Quad (H1)
- DMD Quad (H1)
- Heart (H3)
- Skel Muscle (H3)
- Non-Muscle (H3)
- Norm Skel Muscle, Pediatric (H2)
- Norm Skel Muscle, Older (H2)

| Sample | P value | Sample | P value |
|---|---|---|---|
| DEL2 | 0.0001 | DEL5 | 0.0001 |
| GAS2 | *0.2029* | GAS5 | 0.0004 |
| QUD2 | *0.2183* | QUD5 | 0.0001 |
| TA2 | 0.0001 | TA5 | 0.0001 |
| DEL3 | 0.0001 | DEL6 | 0.0045 |
| GAS3 | 0.0001 | GAS6 | 0.0001 |
| QUD3 | 0.0001 | QUD6 | 0.0002 |
| TA3 | 0.0001 | TA6 | 0.0022 |
| DEL4 | 0.0001 | DEL7 | *0.1163* |
| GAS4 | 0.0001 | GAS7 | 0.0001 |
| QUD4 | 0.0001 | QUD7 | 0.0058 |
| TA4 | 0.0001 | TA7 | 0.0004 |
| | | DEL8 | 0.0001 |
| | | GAS8 | *0.2346* |
| | | QUD8 | 0.0001 |
| | | TA8 | 0.0001 |
| | | DEL9 | 0.0011 |
| | | GAS9 | 0.0003 |
| | | QUD9 | 0.0058 |
| | | TA9 | 0.0002 |
