# Supplementary material for: Transcriptome-scale similarities between mouse and human skeletal muscles with normal and myopathic phenotypes
Source: BMC Musculoskelet Disord. 2006 Mar 7;7:23. doi: 10.1186/1471-2474-7-23 (PMC1525166; doi:10.1186/1471-2474-7-23)
Supplement: Additional File 5 — Adobe pdf file. Correlations of human transcriptome profiles relative to homologous mouse muscle profiles. Here, all samples are characterized by their 271-gene profile of dominant contributors to sample variance in the mouse dataset M (i.e., genes with absolute loadings in PC1-3 exceeding 0.03 (ST2). [file 1471-2474-7-23-S5.pdf]

Supp Figure 5 271-gene mouse muscle sub-transcriptome |PC1-3| > 0.03 (ST2) correlations

A

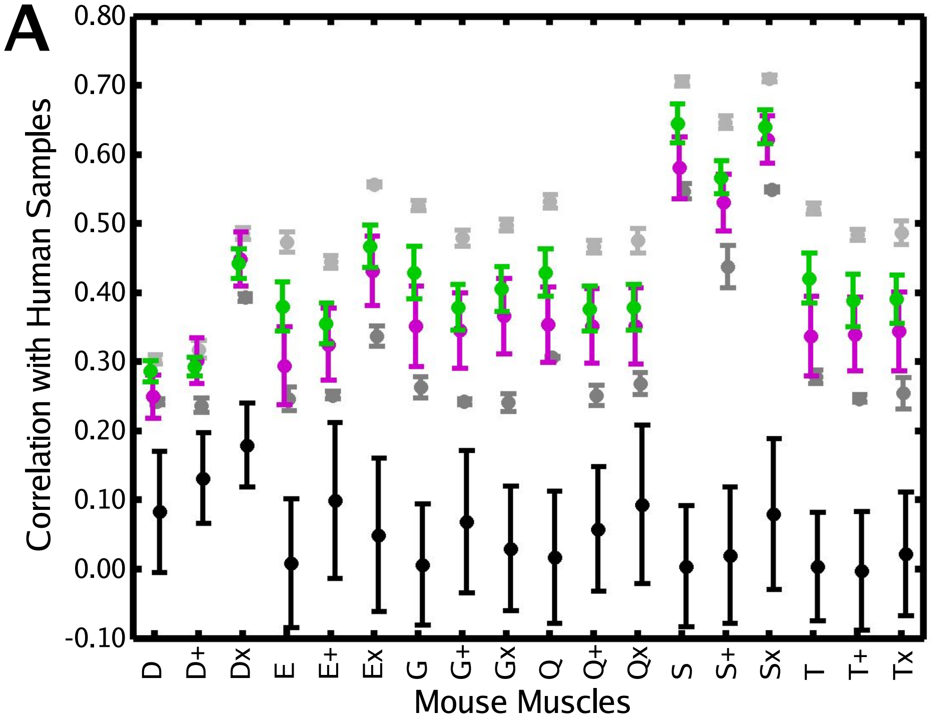

C

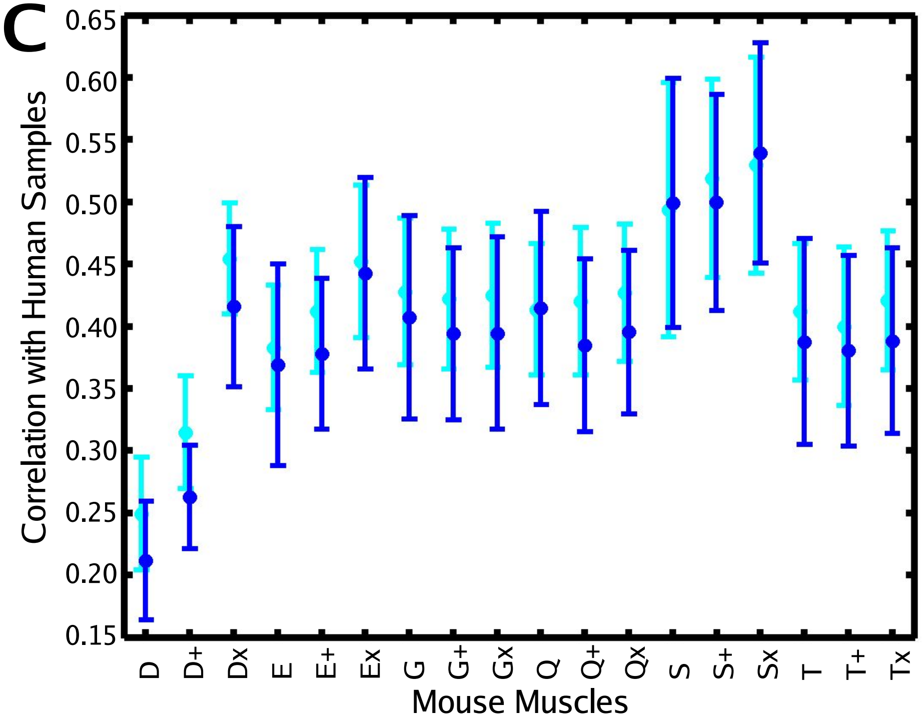

B

Sample	P value	Sample	P value	Sample	P value
Con_169	0.0001	DMD_22	0.0001	BoneMarrow1	*0.7342*
Con_130	0.0001	DMD_21	0.0004	BoneMarrow2	*0.8319*
Con_180	0.0001	DMD_79	0.0001	Brain1	*0.0818*
Con_181	0.0001	DMD_798	0.0001	Brain2	*0.0978*
Con_147	0.0001	DMD_793	0.0001	Kidney1	*0.2183*
Con_140	0.0001	DMD_791	0.0001	Kidney2	*0.2029*
Con_141	0.0001	DMD_823	0.0001	Liver1	*0.1067*
Con_145	0.0001	DMD_251	0.0001	Liver2	*0.1265*
Con_146	0.0001	DMD_45	0.0001	Lung1	*0.5811*
Con_148	0.0001	DMD_825	0.0001	Lung2	*0.5811*
Con_142	0.0001	DMD_87	0.0001	Pancreas1	*0.9323*
Con_144	0.0001	DMD_878	0.0001	Pancreas2	*0.8652*
				Prostate1	*0.2698*
		Heart1	0.0001	Prostate2	*0.2183*
		Heart2	0.0001	SpinalCord1	*0.2698*
		SkelMusc1	0.0001	SpinalCord2	*0.2886*
		SkelMusc2	0.0001	Spleen1	*0.4970*
				Spleen2	*0.5523*
Wilcoxon P value				Thymus1	*0.8987*
SOL vs. non-SOL correl				Thymus2	*0.9661*

D

Color Code (Dataset)
Norm Quad (H1)
DMD Quad (H1)
Heart (H3)
Skel Muscle (H3)
Non-Muscle (H3)
Norm Skel Muscle, Pediatric (H2)
Norm Skel Muscle, Older (H2)

Sample	P value	Sample	P value
DEL2	0.0002	DEL5	0.0001
GAS2	*0.1882*	GAS5	0.0002
QUD2	*0.0509*	QUD5	0.0001
TA2	0.0026	TA5	0.0001
DEL3	0.0001	DEL6	0.0005
GAS3	0.0001	GAS6	0.0001
QUD3	0.0001	QUD6	0.0001
TA3	0.0001	TA6	0.0001
DEL4	0.0196	DEL7	0.0005
GAS4	0.0002	GAS7	0.0001
QUD4	0.0034	QUD7	0.0007
TA4	0.0008	TA7	0.0001
		DEL8	0.0001
		GAS8	*0.1163*
		QUD8	0.0001
		TA8	0.0001
		DEL9	0.0001
		GAS9	0.0001
		QUD9	0.0004
		TA9	0.0001
