# Supplementary material for: Transcriptome-scale similarities between mouse and human skeletal muscles with normal and myopathic phenotypes
Source: BMC Musculoskelet Disord. 2006 Mar 7;7:23. doi: 10.1186/1471-2474-7-23 (PMC1525166; doi:10.1186/1471-2474-7-23)
Supplement: Additional File 6 — Adobe pdf file. Correlations of human transcriptome profiles relative to homologous mouse muscle profiles. Here, all samples are characterized by their 234-gene profile of dominant contributors to sample variance in the human dataset H1 (i.e., genes with absolute loadings in PC1-3 exceeding 0.03 (ST2). [file 1471-2474-7-23-S6.pdf]

Supp Figure 6 234-gene human muscle sub-transcriptome |PC1-3| > 0.03 (ST3) correlations

A

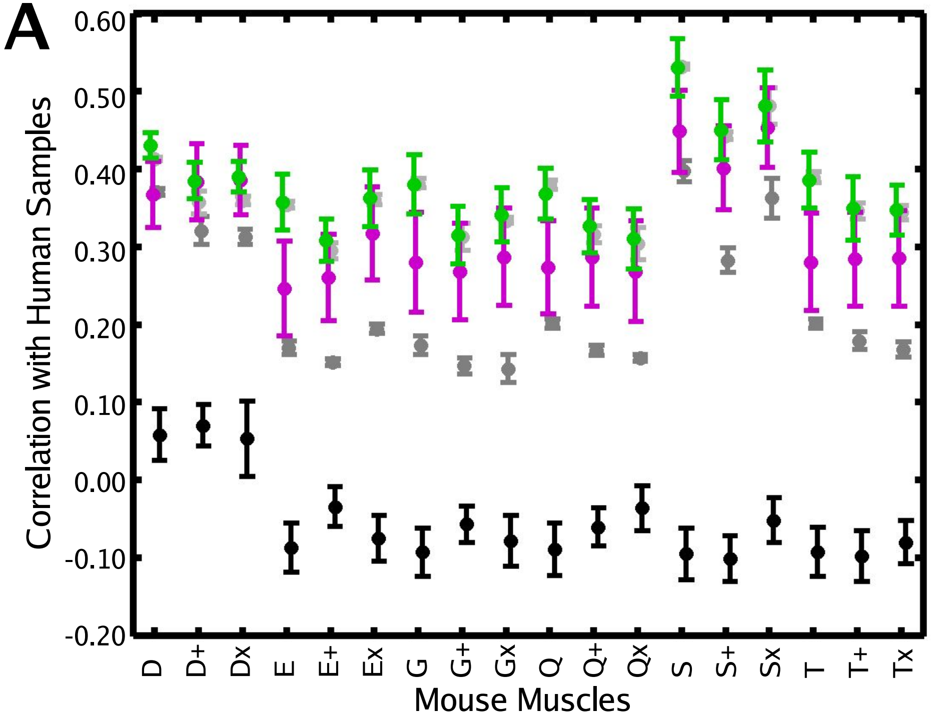

C

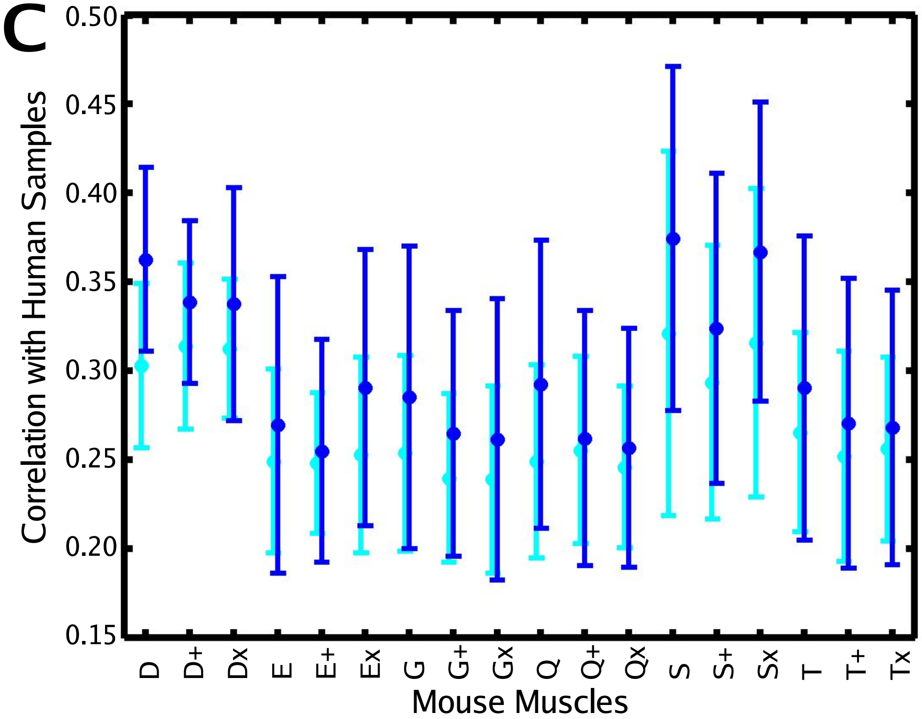

B

Sample	P value	Sample	P value	Sample	P value
Con_169	0.0001	DMD_22	0.0001	BoneMarrow1	*0.1613*
Con_130	0.0001	DMD_21	0.0058	BoneMarrow2	*0.2518*
Con_180	0.0002	DMD_79	0.0002	Brain1	0.0017
Con_181	0.0001	DMD_798	0.0008	Brain2	0.0080
Con_147	0.0001	DMD_793	0.0002	Kidney1	*0.2518*
Con_140	0.0002	DMD_791	0.0002	Kidney2	*0.1265*
Con_141	0.0001	DMD_823	0.0002	Liver1	*0.4970*
Con_145	0.0002	DMD_251	0.0002	Liver2	*0.4970*
Con_146	0.0002	DMD_45	0.0001	Lung1	*0.2183*
Con_148	0.0002	DMD_825	0.0003	Lung2	*0.2029*
Con_142	0.0001	DMD_87	0.0001	Pancreas1	*0.3504*
Con_144	0.0058	DMD_878	0.0002	Pancreas2	*0.4200*
				Prostate1	*0.6712*
		Heart1	0.0013	Prostate2	*0.7342*
		Heart2	0.0013	SpinalCord1	0.0155
		SkelMusc1	0.0001	SpinalCord2	0.0174
		SkelMusc2	0.0001	Spleen1	*0.2028*
				Spleen2	*0.2518*
Wilcoxon P value				Thymus1	*0.2029*
SOL vs. non-SOL correl				Thymus2	*0.1882*

D

Color Code (Dataset)
Norm Quad (H1)
DMD Quad (H1)
Heart (H3)
Skel Muscle (H3)
Non-Muscle (H3)
Norm Skel Muscle, Pediatric (H2)
Norm Skel Muscle, Older (H2)

Sample	P value	Sample	P value
DEL2	0.0045	DEL5	0.0002
GAS2	*0.3504*	GAS5	0.0075
QUD2	*0.7342*	QUD5	0.0001
TA2	*0.0746*	TA5	0.0002
DEL3	0.0001	DEL6	0.0416
GAS3	0.0002	GAS6	0.0096
QUD3	0.0001	QUD6	0.0304
TA3	0.0001	TA6	0.0051
DEL4	*0.8319*	DEL7	0.0245
GAS4	0.0039	GAS7	0.0085
QUD4	*0.0978*	QUD7	0.0273
TA4	0.0123	TA7	0.0058
		DEL8	0.0003
		GAS8	0.0019
		QUD8	0.0002
		TA8	0.0002
		DEL9	0.0004
		GAS9	0.0002
		QUD9	*0.7664*
		TA9	0.0004
